# Supplementary material for: Bioaerosol Sampling Devices and Pretreatment for Bacterial Characterization: Theoretical Differences and a Field Experience in a Wastewater Treatment Plant
Source: Microorganisms. 2024 May 10;12(5):965. doi: 10.3390/microorganisms12050965 (PMC11124041; doi:10.3390/microorganisms12050965)
Supplement: Supplementary file 1 [file microorganisms-12-00965-s001.zip › Figure S1_Table S1, S2.pdf]

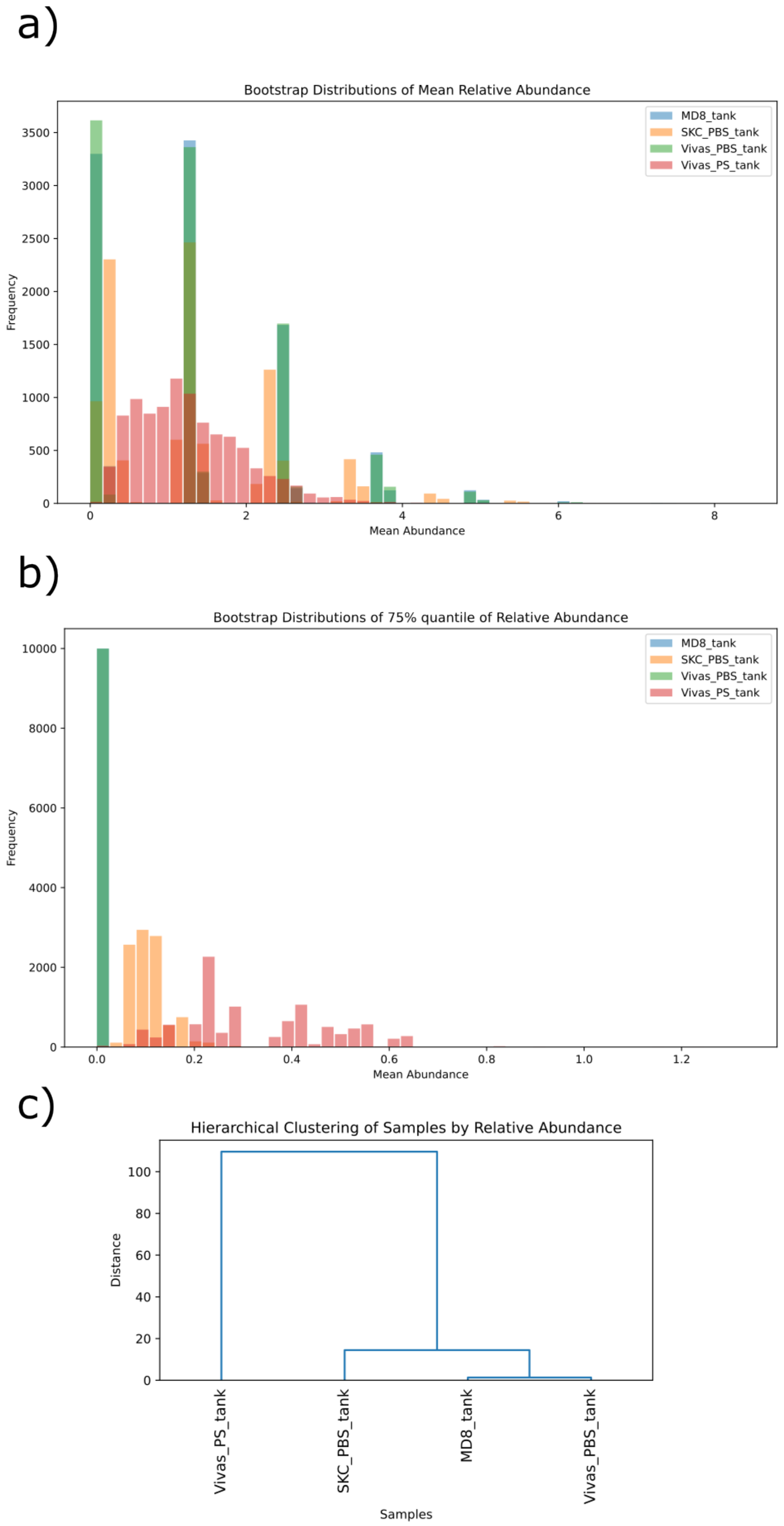

Supplementary Figure S1: Analysis of the differences between samples via bootstrap (n = 10000) distribution of mean (a) relative abundance and upper quartile (75th percentile, b) of the same value. Both figures show a much more even distribution for sample Vivas PS tank, highlighting the significantly higher genus diversity compared to the other samples. Panel (c) shows the dendrogram relative to hierarchical clustering of relative abundance, based on complete linkage of Euclidean pairwise distances. The air samples collected using the Airport MD8 has as sample ID: “MD8_tank”. The air sample collected using the BioSampler has as sample ID: “SKC_PBS_tank”. The air samples collected using the BioSpot-VIVAS have as sample ID: “Vivas_PBS_tank” (sampled using the PBS as collection media) and “Vivas_PS_tank” (sampled using the preserving solution of nucleic acids DNA/RNA shield™ as collection media).

Supplementary Table S1: Recent studies on non culturable and culturable bacteria characterization by 16S rRNA gene sequencing on bioaerosol from WWTPs.

**Paper**	**Sampler**	**Volume per sample**	**Location**	**Quantitative results**	**Qualitative results (predominant genera)**
Hubad, B., & Lapanje, A. (2013). The efficient method for simultaneous monitoring of the culturable as well as nonculturable airborne microorganisms. *PloS one*, 8(12), e82186. https://doi.org/10.1371/journal.pone.0082186	RCS High Flow air samplers (Biotest, Germany). 100 lpm	2000 L Impaction holders for culture independent analysis/ 500 L for culturable bacteria	WWTP and alpine site in Slovenia	Bioaerosol @ WWTP: 800-2300 CFU/m^3- 16S Gene $4x10^7$:$9x10^8$ Copy numbers/m^3 - Alpine site 47-145 CFU/m^3	High ratio between unculturable and culturable bacteria. Molecular methods highlight pathogens (e.g. *Streptococcus pneumoniae*) or opportunistic pathogens (e.g. *Mycobacterium avium*) absent from culturable bacteria. Method was applied to quantify and to determine diversity of bacteria in air from the WWTP and from alpine environment
Kowalski, M., Wolany, J., Pastuszka, J.S. et al. (2017). Characteristics of airborne bacteria and fungi in some Polish wastewater treatment plants. *Int. J. Environ. Sci. Technol.*, 14, 2181–2192. https://doi.org/10.1007/s13762-017-1314-2	Six-stage Andersen impactor	226,4 L	5 WWTPs in Poland	airborne bacteria and fungi ranged from 10^2 to 10^3 CFU/m^3	The Gram-positive cocci and nonsporing Gram-positive rods were the dominating forms - most antibiotic- resistant features were present among *Bacillus* species (especially *Bacillus mycoides*)
Han, Y., Yang, K., Yang, T., Zhang, M., & Li, L. (2019). Bioaerosols emission and exposure risk of a wastewater treatment plant with A2O treatment process. *Ecotoxicology and environmental safety*, 169, 161–168. https://doi.org/10.1016/j.ecoenv.2018.11.018	Six Stage Viable Andersen Cascade Impactor	84,90 L	Anaerobic-anoxic-oxic (A2O) process, Beijing, China	1.00×10^4 CFU/m^3, summer max	18 of the 65 species of bacteria detected potentially or opportunistically pathogenic, such as *Chryseobacterium*, *Stenotrophomonas*, *Alcaligenes*, *Micrococcus*, *Pantoea*, *Enterobacter* and *Escherichia-Shigella*
Gaviria-Figueroa, A., Preisner, E. C., Hoque, S., Feigley, C. E., & Norman, R. S. (2019). Emission and dispersal of antibiotic resistance genes through bioaerosols generated during the	PTFE filter (culture independent analysis); Airport	5040 L for culture-independent analysis.	WWTP along the coast of South Carolina	logarithm of the antibiotic resistant gene copies per ng	*Zoogloea*, *Thauera*, *Azospira*, *Dechloromonas*, *Chitinivorax*, *Candidatus Accumulibacter*, *Rhodobacter*, *Alkanindiges*,

treatment of municipal sewage. *The Science of the total environment*, 686, 402–412. https://doi.org/10.1016/j.scitotenv.2019.05.454	MD8 (culture dependent analysis)	3750 L for culture-dependent microbial community and ARG analysis		of DNA (amount of DNA?)	*Acinetobacter*, *Candidatus Competibacter*, *Flavobacterium*, *Ferruginibacter*, *Leadbetterella*, *Nitrospira*
Wan, J.; Zhang, Z.; Huo, Y.; Wang, X.; Wang, Y.; Wu, J.; Huo, M. Particle Size Matters: Distribution, Source, and Seasonality Characteristics of Airborne and Pathogenic Bacteria in Wastewater Treatment Plants (2023). *Atmosphere*, 14, 465. https://doi.org/10.3390/atmos14030465	Eight-stage Andersen impactor	13.584 L (culture independent analysis)	2 WWTP in Changchun, China	92 to 1793 ASV per sample	Bacillales, Mycobacterium, and Pseudomonas in winter; Brevundimonas in autumn; and Acinetobacter, Comamonas, and Ralstonia in summer.
Yang, T., Han, Y., Liu, J., & Li, L. (2019). Aerosols from a wastewater treatment plant using oxidation ditch process: Characteristics, source apportionment, and exposure risks. *Environmental pollution*, 250, 627–638. https://doi.org/10.1016/j.envpol.2019.04.071	TSP 100 L/min over a period of 4 h for culture independent analysis; Six-stages Andersen impactors 2 min at a flow rate of 28.3 L/min for culturable bacteria	24000 L per culture independent analysis / 56,6 L per culturable bacteria	WWTP Hefei, China	Up to 3807 CFU/m^3	Geothrix, Microthrix, Cyanobacteria, Saprospiraceae, Chroococcidiopsis (among pathogrenic b.:Rhodococcus, Mycobacterium and Arcobacter)
This paper	Airport MD8; SKC Biosampler; BioSpot ViVAS	1440 L (culture independent analysis)	WWTP Trieste (Italy) - this study	Bioaerosol: 16 101 of trimmed reads and 64 OTUs	Biospot Vivas_PS: Klebsiella, Enterobacter, Pseudomonas, and Acinetobacter; SKC Biosampler and Airport MD8: Stenotrophomonas

Supplementary Table S2: Number of trimmed reads used for each sample.

	sample	Sampling	number of trimmed reads
**bioaerosol samples**	SKC_PBS_tank	BioSampler	3375
	Vivas_PBS_tank	BioSpot-VIVAS	4471
	Vivas_PS_tank	BioSpot-VIVAS	5096
	MD8_tank	Airport MD8	3159
**wastewater samples**	Water_1	Grab	6075
	Water_2	Grab	6917
	Water_24h_1	Composite	5650
	Water_3	Grab	7407
	Water_24h_2	Composite	7456
	Water_4	Grab	10750
	Water_24h_3	Composite	6139
	Water_5	Grab	5544
	Water_24h_4	Composite	6180
	Water_6	Grab	5523
	Water_7	Grab	2446
